# Osteogenic cells form mineralized particles, a few *μ*m in size, in a 3D collagen gel culture

**DOI:** 10.7717/peerj.7889

**Published:** 2019-10-23

**Authors:** Takanori Kihara, Chiya Umezu, Karin Sawada, Yukari Furutani

**Affiliations:** Department of Life and Environment Engineering, Faculty of Environmental Engineering, The University of Kitakyushu, Kitakyushu, Fukuoka, Japan

**Keywords:** Mineralized matrices, Osteogenic cells, Collagen gel, Mineralized particles, Calcospherulites, 3D culture

## Abstract

Osteogenic cells form mineralized matrices in vitro, as well as in vivo. The formation and shape of the mineralized matrices are highly regulated by the cells. In vitro formation of mineralized matrices by osteogenic cells can be a model for in vivo osteogenesis. In this study, using a three-dimensional (3D) collagen gel culture system, we developed a new in vitro model for the formation of mineralized particles, a few µm in size, by the osteogenic cells. Human osteosarcoma (HOS) cells formed spherical mineralized matrices (about 12 µm) at approximately 7 days when cultured with β-glycerophosphate (β-GP)-containing culture media on 2D tissue culture plates. Alternately, when they were cultured in a 3D collagen gel containing β-GP, they formed mineralized particles with about 1.7 µm in the gel at approximately 3 days. Calcium precipitation in the gel was evaluated by measuring the gel turbidity. This type of mineralization of HOS cells, which formed mineralized particles inside the gel, was also observed in a peptide-based hydrogel culture. The mineralized particles were completely diminished by inhibiting the activity of Pit-1, phosphate cotransporter, of the HOS cells. When mouse osteoblast-like MC3T3-E1 cells, which form large and flat mineralized matrices in 2D osteogenic conditions at approximately 3 weeks of culture, were cultured in a 3D collagen gel, they also formed mineralized particles in the gel, similar to those in HOS cells, at approximately 18 days. Thus, osteogenic cells cultured in the 3D collagen gel form mineralized particles over a shorter period, and the mineralization could be easily determined by gel turbidity. This 3D gel culture system of osteogenic cells acts as a useful model for cells forming particle-type mineralized matrices, and we assume that the mineralized particles in the 3D hydrogel are calcospherulites, which are derived from matrix vesicles secreted by osteogenic cells.

## Introduction

Mineralized matrices consist of organic extracellular components, such as collagen fibers and inorganic hydroxyapatite crystals (Veis, 2003). The formation and shape of mineralized matrices *in vivo* are highly regulated by osteogenic cells. This requires the concerted action of several microenvironmental signals, including cytokines, extracellular matrices, and cell–cell interactions (Lian & Stein, 1992). On the other hand, osteogenic cells also form mineralized matrices in vitro. Physicochemical and biochemical analyses of this in vitro mineralization show that the mineralized matrix is not a simple precipitation of calcium phosphate, but the result of a biological process, because the mineralized matrix contains apatite crystals and bone matrix proteins (Kale et al., 2000; Kihara et al., 2004; Maniatopoulos, Sodek & Melcher , 1988; Ohgushi et al., 1996). Thus, in vitro formation of mineralized matrices by osteogenic cells can be used as a model for studying in vivo osteogenesis.

There are many in vitro osteogenesis models, using several types of osteogenic cells (Czekanska et al., 2012). In particular, human osteosarcoma (HOS) cells (Sonobe et al., 1991), Saos-2 cells (Rodan et al., 1987), mouse osteoblast-like MC3T3-E1 cells (Sudo et al., 1983), rat calvarial osteoblasts (Declercq et al., 2004), and rat mesenchymal stem cells (Kihara et al., 2006; Kihara et al., 2004; Maniatopoulos, Sodek & Melcher , 1988) are commonly used osteogenic cells in vitro. In this study, we used HOS and MC3T3-E1 cells. HOS cells, an osteoblastic cell line derived from malignant tumors of mesenchymal cells, produce a prominent osteoid with mineralization (Sonobe et al., 1991). HOS cells cultured with β-glycerophosphate (β-GP) begin forming mineralized matrices at approximately 5 days (Uchimura et al., 2003). MC3T3-E1 cells, a clonal preosteoblastic cell line derived from newborn mouse calvaria, form an abundant collagen matrix and also show mineralization (Al-Jallad et al., 2006; Sudo et al., 1983). When cultured with β-GP, ascorbic acid, and dexamethasone, MC3T3-E1 cells start to form mineralized matrices at approximately 3 weeks (Kihara, 2015). These cells have been demonstrated as useful in vitro models for studying osteoblastic differentiation and mineralization (Czekanska et al., 2012; Kartsogiannis & Ng, 2004; Kotobuki et al., 2008; Sonobe et al., 1991; Uchimura et al., 2003). Both cells form mineralized matrices under osteogenic conditions, but the shapes of these mineralized matrices are totally different; HOS cells form many spherical mineralized matrices, and MC3T3-E1 cells form large and flat mineralized matrices (Kihara, 2015). The formed mineralized matrices vary according to the osteogenic cell type.

Three-dimensional (3D) collagen gel culture is used as a model for mimicking the *in vivo* tissue environment. Dermal fibroblasts cultured in collagen gels are specifically used in the artificial dermis for regenerative medicine (Bell, Ivarsson & Merrill, 1979). Furthermore, chondrocytes cultured in collagen gel are used as regenerative artificial cartilage (Ochi et al., 2002). Osteogenic cells cultured in 3D collagen gels show the upregulation of osteogenic-related gene expression, deposition of mineralized matrices, and osteocyte-like morphology (Lund, Stegemann & Plopper , 2009; Murshid et al., 2007; Parreno et al., 2008; Yoneno et al., 2005). Thus, 3D collagen culture of osteogenic cells is a critical tool for accelerating bone regeneration in bone defects and studying *in vivo* osteogenesis. However, few studies have investigated the mineralized matrices formed by osteogenic cells in 3D collagen gels.

In this study, we focused on the shape of mineralized matrices formed by osteogenic cells in 3D collagen gels. HOS cells formed mineralized particles, around 1.7 µm in size, inside the collagen gel, thereby decreasing gel transparency. The mineralized particles were also formed in a 3D peptide hydrogel culture of HOS cells. Furthermore, MC3T3-E1 cells formed similar mineralized particles in a 3D collagen gel culture. Our results show that osteogenic cells commonly form mineralized particles, a few µm in size in a 3D collagen gel culture with apparently different shapes from the mineralized matrices formed under 2D culture.

## Materials and Methods

### Materials

Human osteosarcoma HOS and mouse osteoblast-like MC3T3-E1 cells were obtained from the Health Science Research Resources Bank (Osaka, Japan). Antibiotics and G418 were purchased from Sigma-Aldrich (St. Louis, MO). Glass-based culture dishes were purchased from Matsunami Glass (Osaka, Japan). Type I collagen (acid extract from porcine tendon) solution was purchased from Nitta Gelatin Co. (Osaka, Japan). Cell-removing solution (TrypLE express) was purchased from Life Technologies Japan Ltd. (Tokyo, Japan). pEYFP-C1 plasmid encoding an enhanced yellow fluorescence protein (EYFP) was purchased from Clontech (Mountain View, CA). FuGENE HD was purchased from Roche Diagnostics (Basel, Switzerland). Panacea Gel was purchased from Menicon Life Science (Aichi, Japan). The calcium E-test and Foscarnet were purchased from Wako Pure Chemical Industries Ltd. (Osaka, Japan). Other reagents were purchased from Sigma-Aldrich, Wako Pure Chemical Industries Ltd., or Life Technologies Japan Ltd.

### Cell culture and mineralization induction

HOS and MC3T3-E1 cells were cultured in Dulbecco’s modified Eagle medium (DMEM) containing 10% fetal bovine serum (FBS) and antibiotics (100 units/mL penicillin G and 100 µg/mL streptomycin sulfate) at 37 °C under a humidified atmosphere of 5% CO_2_ and 95% air. The cells were seeded in culture plates at 1.0 × 10^4^ cells/cm^2^. The culture medium was replaced twice a week. To induce mineralization in HOS cells, the cells were cultured in a medium supplemented with 10 mM β-GP. To induce mineralization in MC3T3-E1 cells, the cells were cultured in a medium supplemented with 82 µg/mL ascorbic acid-2-phosphate, 10 mM *β*-GP, and 10 nM dexamethasone (Kihara, 2015).

### Alizarin red S staining for mineralization

The mineralized matrices were stained by Alizarin red S. The cultured cells were firstly washed with PBS and fixed with 4% paraformaldehyde. After washing, the fixed cells were stained with 0.1% Alizarin red S solution for 10 min, rinsed and observed microscopically.

### Construction of HOS cells stably expressing EYFP

HOS cells stably expressing EYFP were constructed. Firstly, HOS cells were transfected with pEYFP-C1 plasmid using the FuGENE HD transfection reagent, as recommended by the manufacturer. The transfected cells were cultured and screened in the neomycin analog G418-containing medium. The cells expressing neomycin resistance gene encoded in pEYFP-C1 plasmid can be grown under the G418-containing medium. The EYFP-expressing cells were manually picked up while under observation via fluorescence microscopy. The selected cells were cultured and passaged several times in a G418-containing medium. Finally, we obtained HOS cells stably expressing EYFP (HOS-EYFP cells).

### Preparation of hydrogel-containing cells

Under physiological conditions (37 °C, neutral pH in saline), type I collagen has self-assembling activity; at a high collagen concentration, the collagen solution becomes a gel (Hayashi, 1978). Artificial peptide-based Panacea Gel solution becomes a gel by mixture with equal volume of medium and incubation at 37 °C.

We used these hydrogels to embed cultures of osteogenic cells. The cells were removed from dishes by treatment with TrypLE express, cell-removing solution, washed with the culture medium, counted using a hemocytometer, and suspended at an appropriate density. Type I collagen solution containing these cells in the culture medium was prepared by mixing the collagen solution (3 mg/mL) with 5 × concentrated DMEM (phenol red-free), 10% FBS, and the cell suspension at 4 °C resulting in a final collagen concentration of 1.9 mg/mL and cell density of 5.0 × 10^5^ cells/mL. The cell-containing collagen solution was plated, and the culture plates were incubated for 30 min at 37 °C for gelation. The Panacea Gel was mixed with the cells as recommended by the manufacturers and then plated. After gelation, the culture media were added onto the gel and the cells were cultured. The calcification of the hydrogel was evaluated by examining changes in gel turbidity absorption at wavelength of 750 nm.

### Fluorescence observation

For fluorescence observation, the culture medium was supplemented with 1 µg/mL calcein or xylenol orange, fluorescent dyes with affinity for calcium ion, which are incorporated into the precipitated calcium (Hirose et al., 2003; Kihara et al., 2006; Kihara et al., 2004; Uchimura et al., 2003). The specimens were observed by fluorescence microscopy (IX-81; Olympus, Tokyo, Japan or BZ-9000; Keyence, Osaka, Japan) or confocal laser scanning microscopy (CLSM) (Nikon C2; Nikon, Tokyo, Japan) using a 60 × oil immersion lens (NA = 1.42). Serial sections (1 µm pitch) of specimens were obtained and the serial images were superimposed using ImageJ software (NIH, Bethesda, MD). The sizes of mineralized matrices were also analyzed using ImageJ software and shown as a frequency distribution. Peak positions were determined by fitting with the Gaussian distribution.

### Measurement of calcium content of collagen gel

HOS cell-containing collagen solution (100 µL) was plated in each well of 96-well plates, and the culture plates were incubated for 30 min at 37 °C for gelation before addition to the culture medium. The cell-containing gel turbidity was measured at days 1, 2, 3, 4, 5, 6, and 7, then the gel samples were collected daily and centrifuged at 13, 000 × g for 10 min at 4 °C, after which the supernatant was removed. The precipitate was treated with formic acid (30 µL) and incubated at 4 °C for 24 h. Then, the dissolved calcium contents of the supernatant were measured via the Calcium E-test (Wako) according to the manufacturer’s instructions.

### Statistical analysis

The data for each group was statistically compared using the non-parametric Mann–Whitney *U* test. The calcium contents were compared using one-way analysis of variance and Dunnet pairwise comparison test. *p*-values <0.05 were considered statistically significant.

## Results

### HOS cells formed calcium particles, a few µm in diameter, in 3D collagen gel

**Figure 1:**
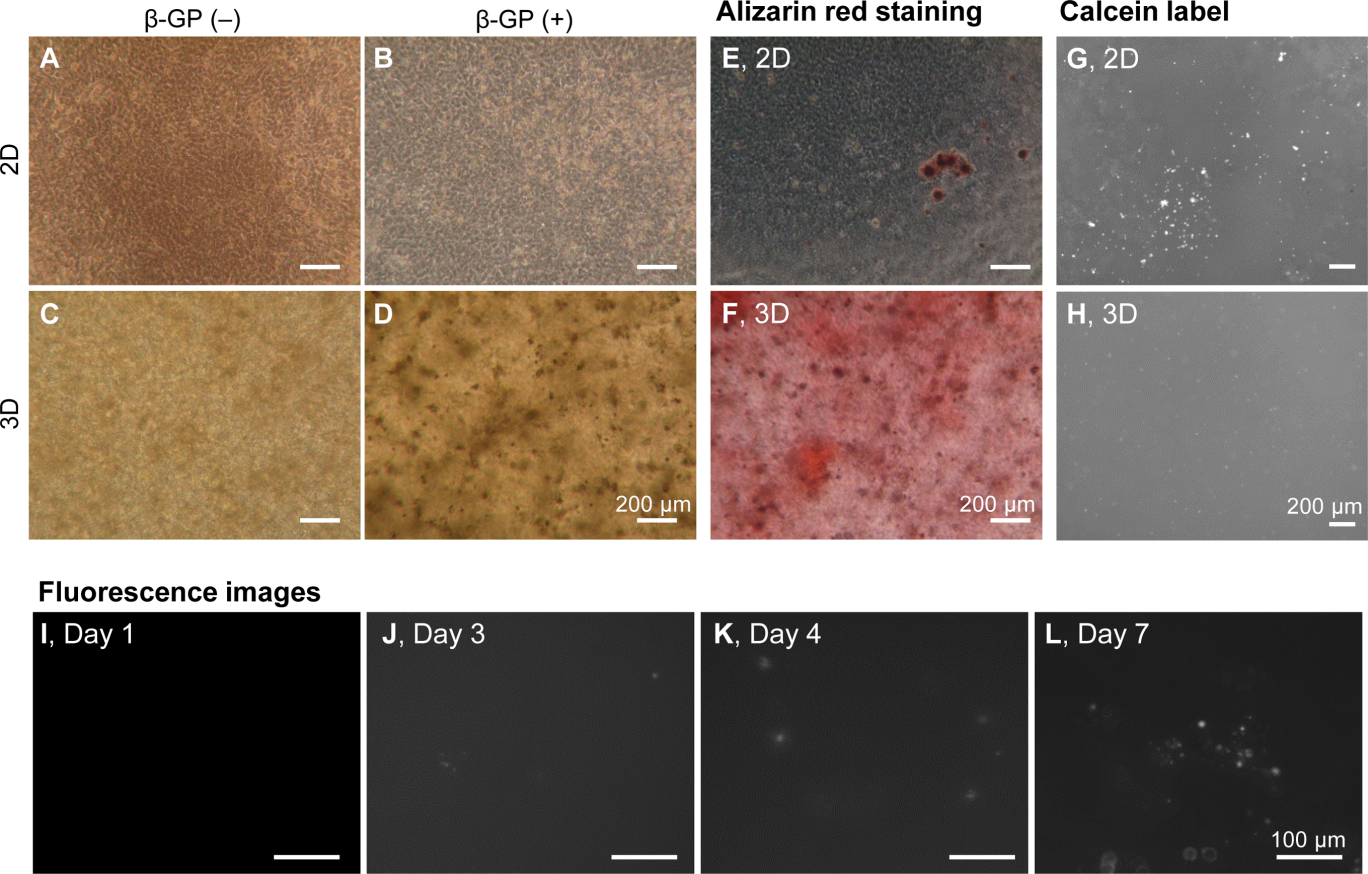
Mineralization of HOS cells in a 3D collagen gel. (A–D) Phase contrast microscopy images of mineralization of HOS cells. HOS cells were cultured with (B, D) or without (A, C) β-GP on 2D conventional culture plates (A, B) or in 3D collagen gel (C, D) for 7 days. Bar: 200 µm. (E, F) Alizarin red S staining images of the mineralized HOS cells. HOS cells were cultured with β-GP on 2D culture plates (E) or in 3D collagen gels (F) for 7 days and then stained with Alizarin red S. Bar: 200 µm. (G, H) Fluorescence images of mineralized HOS cells. HOS cells were cultured with β-GP and calcein on 2D culture plates (G) or in 3D collagen gels (H) for 7 days, after which fluorescence of the precipitated calcein was observed. Bar: 200 µm. (I–L) The formation process of calcium particles by HOS cells in a 3D collagen gel. HOS cells were cultured with β-GP and calcein in a 3D collagen gel. Precipitated calcein in the collagen gel was observed with a fluorescence microscope. Bar: 100 µm.

We firstly cultured HOS cells on tissue culture plates and in 3D type I collagen gels (Fig. 1A). HOS cells formed mineralized matrices in the media containing β-GP (Kihara, 2015). On tissue culture plates, HOS cells formed many mineralized spherical matrices at approximately 7 days (Fig. 1B). These mineralized spherical matrices were confirmed by staining with Alizarin red S and labeling with a calcium-binding fluorescent molecule, calcein (Figs. 1E and 1G). The spherical shapes of the mineralized matrices formed by HOS cells were similar to those described in previous reports (Kihara, 2015; Uchimura et al., 2003). When HOS cells were cultured in 3D type I collagen gel containing β-GP, the collagen gel gradually became clouded and many particles were observed in the collagen gel by phase contrast microscopy (Fig. 1D). These particles in the collagen gel were stained using Alizarin red S and labeled using calcein, confirming that the particles were mineralized matrices (Figs. 1F, 1H). These mineralized particles, first observed after 3 days of culture, were scattered and their number gradually increased (Figs. 1I–1L). Thus, in 3D collagen gel, HOS cells formed mineralized particles after a short culture period.

**Figure 2:**
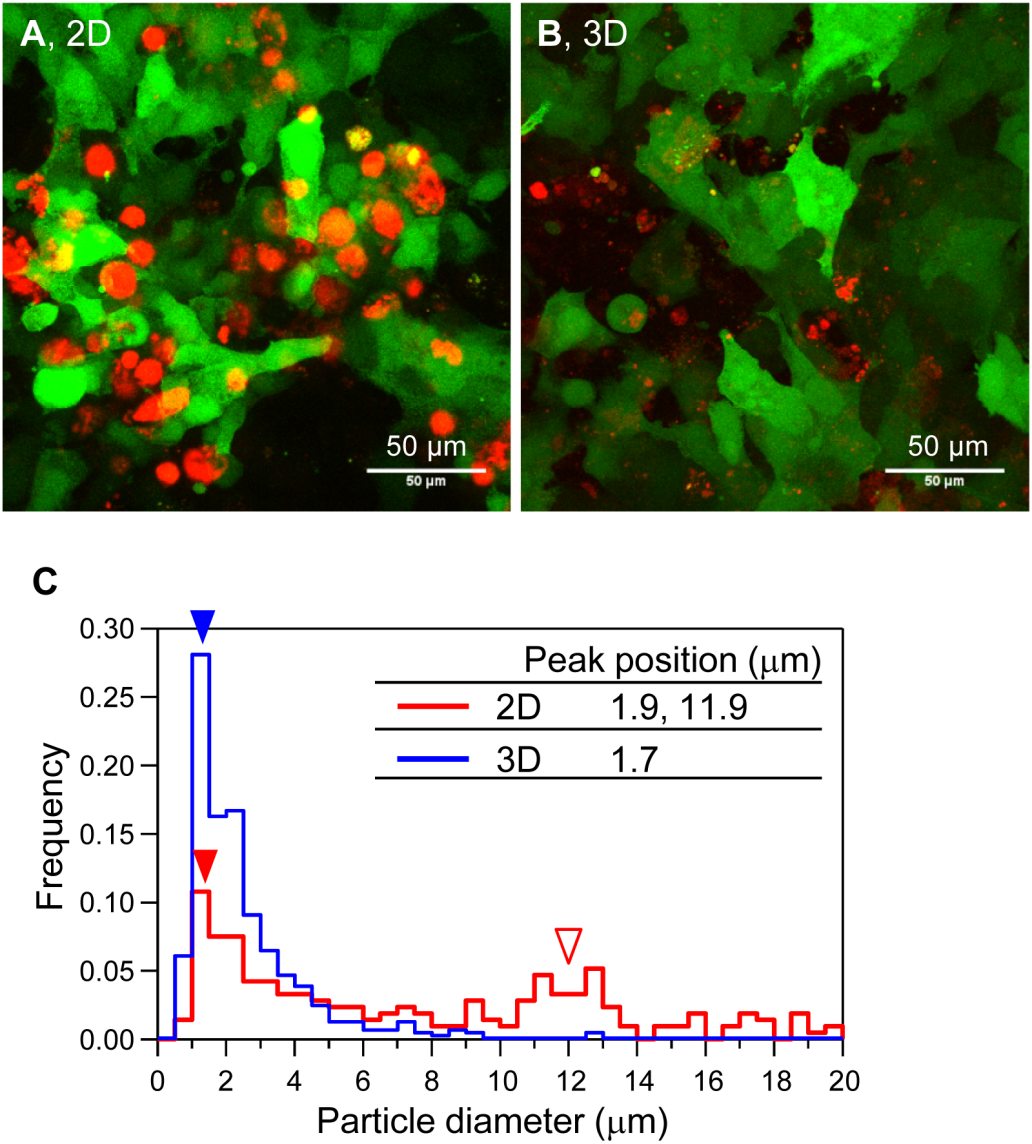
Morphological and size distribution analysis of mineralized matrices of HOS cells. (A, B) Confocal laser scanning microscopy images of the mineralization of HOS-EYFP cells. HOS-EYFP cells were cultured with β-GP and xylenol orange on a 2D glass base dish (A) or in a 3D collagen gel (B) for 7 days. After cell fixing with paraformaldehyde, the cells were observed by a stereoscopic CLSM. The obtained serial images for the *Z*-axis (total 40 µm thickness), were superimposed. Green color shows EYFP fluorescence of HOS-EYFP and red color shows xylenol orange fluorescence. HOS-EYFP cells cultured on a 2D dish formed spherical mineralized matrices (A). Alternately, HOS-EYFP cells cultured in 3D collagen gel formed fine calcium particles around the cells (B). Bar: 50 µm. (C) The size distribution of mineralized matrices formed by HOS-EYFP cells under 2D and 3D culture conditions. The sizes of the deposited mineralized matrices were analyzed using ImageJ software. More than 200 mineralized matrices were analyzed under each condition. Under 2D culture conditions, 2 peaks are shown at 1.9 µm (red fill arrowhead) and ∼11.9 µm (red open arrowhead). On the other hand, under 3D culture conditions, only 1 peak is shown at ∼1.7 µm (blue fill arrowhead).

Next, we observed the shape and size of the mineralized particles of HOS cells in 3D collagen gels by CLSM (Fig. 2). We constructed HOS cells stably expressing EYFP (HOS-EYFP cells) and cultured them in the presence of a calcium-binding red fluorescent molecule, xylenol orange (Hirose et al., 2003; Kihara et al., 2006). The shape of the mineralized matrices formed by HOS-EYFP cells on 2D culture plates was almost spherical, and their size was about 12 µm (Figs. 2A and 2C). The spherical mineralized matrices were located in the cell layer (Fig. 2A). On the other hand, mineralized matrices in the 3D collagen gel were amorphous particles, and their sizes were around 1.7 µm (Figs. 2B and 2C). The mineralized particles were located around the cells (Fig. 2B). Thus, the shapes of mineralized matrices formed by HOS cells on 2D culture plates and in 3D collagen gels were obviously different.

**Figure 3:**
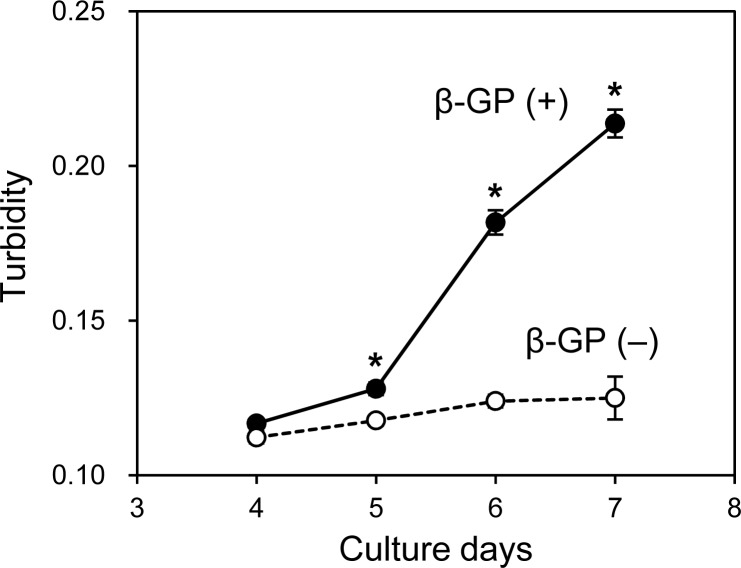
Transition of turbidity of collagen gel embedded with HOS cells. HOS cells were cultured with or without β-GP in 3D collagen gel. The gel volume was 500 μL per well of a 24-well culture plate. The transition of turbidity was measured using a plate reader. *p < 0.05 vs. gel turbidity without β-GP conditions (*N* = 4).

**Figure 4:**
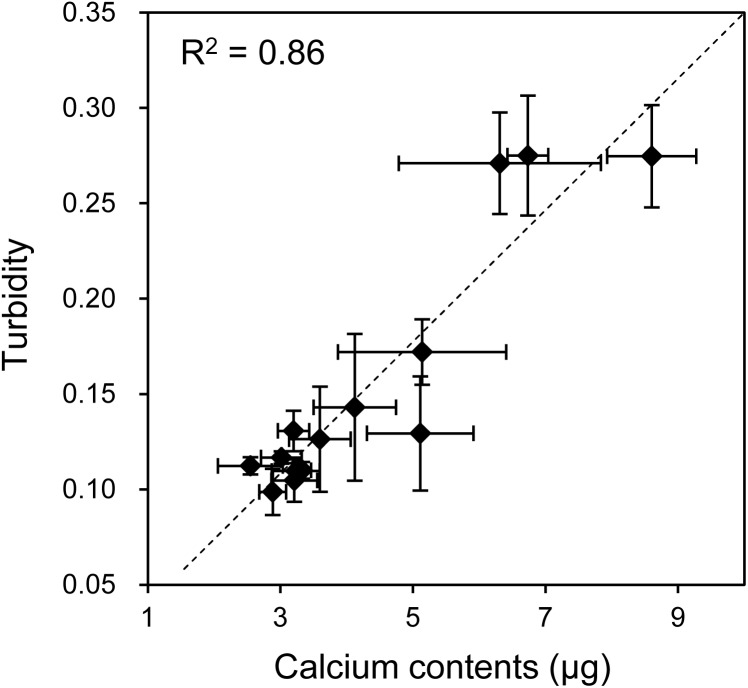
Relationship between the calcium content and turbidity of collagen gel embedded with HOS cells. The turbidities of collagen gel embedding HOS cells were plotted against the calcium contents of the corresponding gels. The calcium was extracted after formic acid treatment of the gels as described in the Materials and Methods. The coefficient of determination (R^2^) is 0.86.

As mentioned, when the HOS cells were cultured in 3D collagen gels with β-GP-containing medium, the gel gradually became clouded. We then measured changes in the collagen gel turbidity (Fig. 3). Although the turbidity of the HOS cells contained in the collagen gel without β-GP was almost constant during the culture period, that of the gels cultured with β-GP gradually increased over time (Fig. 3). Gel turbidity will reflect the amount of mineralized particle precipitation inside the gel. Then, we compared the gel turbidity and the precipitated calcium in the collagen gel (Fig. 4). We found that the gel turbidity and the calcium content were positively correlated (coefficient of determination was 0.86) (Fig. 4), suggesting that the amount and progression of mineralized matrices in 3D collagen gels can be estimated by measuring gel turbidity.

### Mineralization of HOS cells cultured in a peptide-based hydrogel

**Figure 5:**
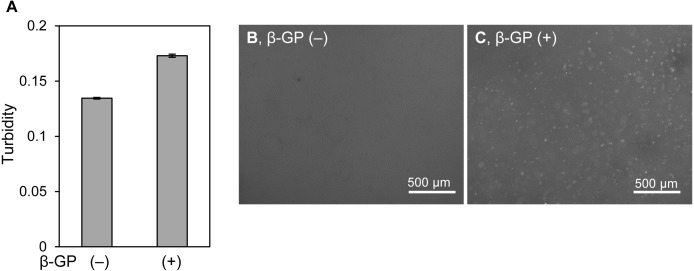
Mineralization of HOS cells in a 3D peptide hydrogel. (A) Turbidity of hydrogels embedding HOS cells. HOS cells were cultured with or without β-GP in a 3D peptide hydrogel, panacea gel, for 6 days. The gel volume was 70 µL per well of a 96-well culture plate. The hydrogel turbidity was measured using a plate reader. The values were calculated from two experiments. (B, C) Fluorescence images of hydrogels embedded with HOS cells. HOS cells were cultured with (C) or without (B) β-GP and with calcein in 3D peptide hydrogels for 6 days, and then the precipitated calcein fluorescence was observed. Bar: 500 µm.

It is unclear why the shapes of mineralized matrices formed by HOS cells on 2D culture plates and in 3D collagen gel are different. The mineralized particles in 3D collagen gels might be affected by the surrounding collagen matrix, because type I collagen can be a crystal nucleus for mineralization (Glimcher, 1976; Hodge, 1989). Then, we cultured HOS cells in another type of 3D hydrogel, Panacea Gel, derived from artificial peptide-containing alginate and aspartic acid with β-GP (Fig. 5) (Nagai et al., 2012). The turbidity of the Panacea Gel, including HOS cells and β-GP, increased after 6 days (Fig. 5A). Mineralized particles were observed inside the gel (Fig. 5C). Thus, the mineralized particles formed by HOS cells in the 3D hydrogel are not specific to a type I collagen matrix, but are a common phenomenon in 3D hydrogels.

### Mineralized particle formation of HOS cells requires membrane transport of inorganic phosphate

**Figure 6:**
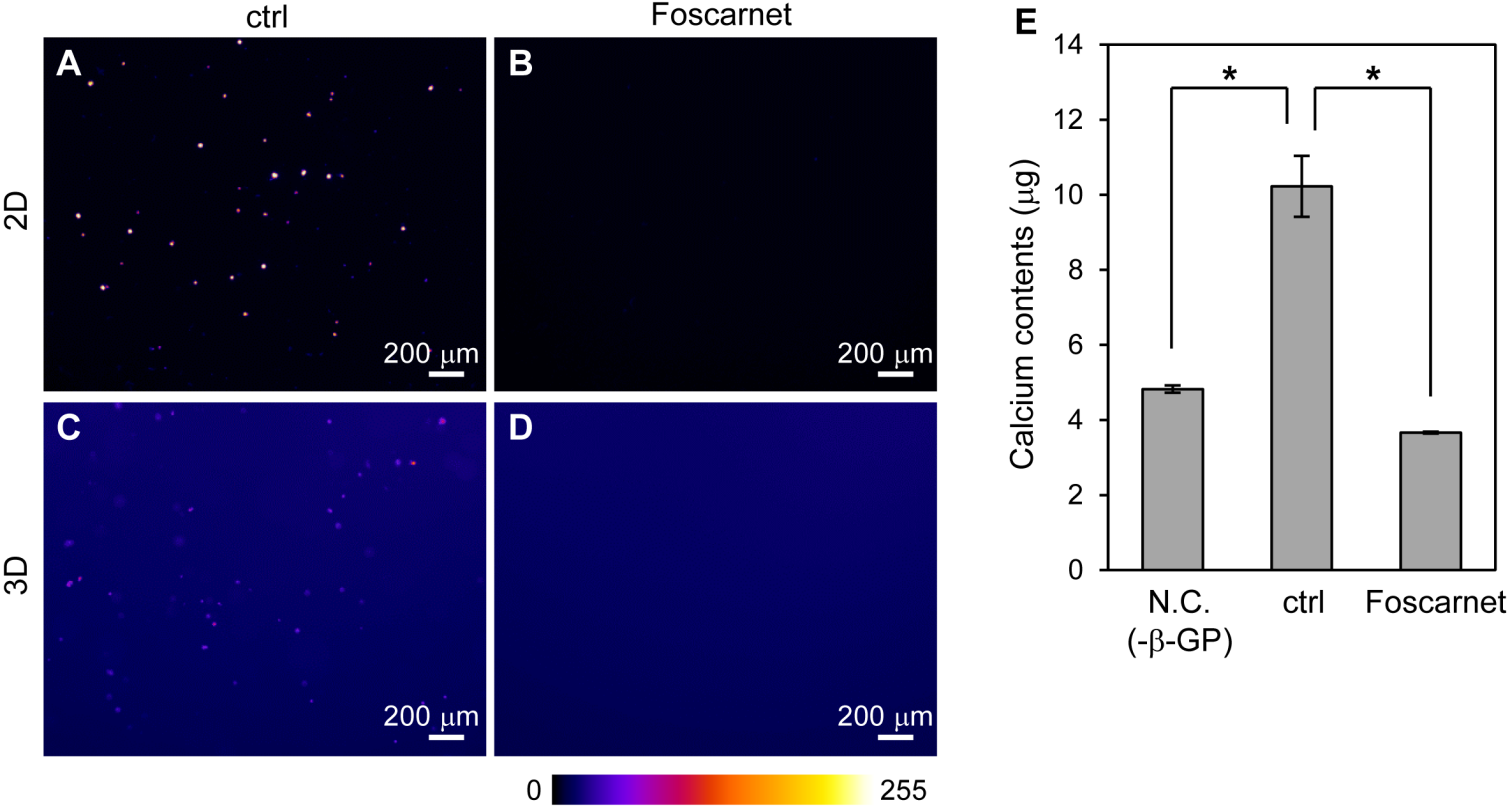
The effect of Foscarnet on the mineralization of HOS cells in a 3D collagen gel. (A–D) Fluorescence images of the HOS cells which were cultured with (B, D) or without (A, C) Foscarnet, an inhibitor of the sodium phosphate transporter, Pit-1, in 2D (A, B) and 3D (C, D) culture conditions for 7 or 8 days. Foscarnet diminished mineralization formation of HOS cells not only 2D but also 3D culture conditions. The fluorescence intensity is represented by a *Fire* color scale. Bar: 200 µm. (E) Calcium contents of the collagen gel embedded with HOS cells. Negative conditions (N.C.) means 3D collagen gel embedded with HOS cells without β-GP. Control conditions (ctrl) means 3D collagen gel embedded with HOS cells with β-GP. Foscarnet conditions means 3D collagen gel embedded with HOS cells with β-GP and Foscarnet. The cells were cultured for 7 days and collected. *p < 0.05 vs. calcium contents of control conditions (Dunnet pairwise comparison test, *N* = 3).

The formation of mineralized particles by HOS cells is a common phenomenon. How do HOS cells form mineralized particles in 3D hydrogel? To examine the possibility that the hydrogel becomes a crystal nucleus, we inhibited the activity of the sodium-dependent phosphate cotransporter, Pit-1, using Foscarnet (Fig. 6) (Yoshiko et al., 2007). By addition of Foscarnet to culture media, mineralized particles formation in the 3D collagen gel and spherical mineralized matrices on the 2D culture plates completely diminished (Figs. 6A–6D). Calcium precipitation in the 3D collagen gel was also completely inhibited (Fig. 6E). Thus, the formation of mineralized particles in the 3D collagen gel requires the membrane transport of inorganic phosphate. That is, mineralization in 3D collagen gels by HOS cells is not a simple calcium phosphate precipitation reaction on collagen fibers, but involves a biological process in the cell membrane. Furthermore, the mineralization mechanism of HOS cells in 3D collagen gel was found to be the same as that on 2D culture plates.

### MC3T3-E1 cells also formed mineralized particles in 3D collagen gel

**Figure 7:**
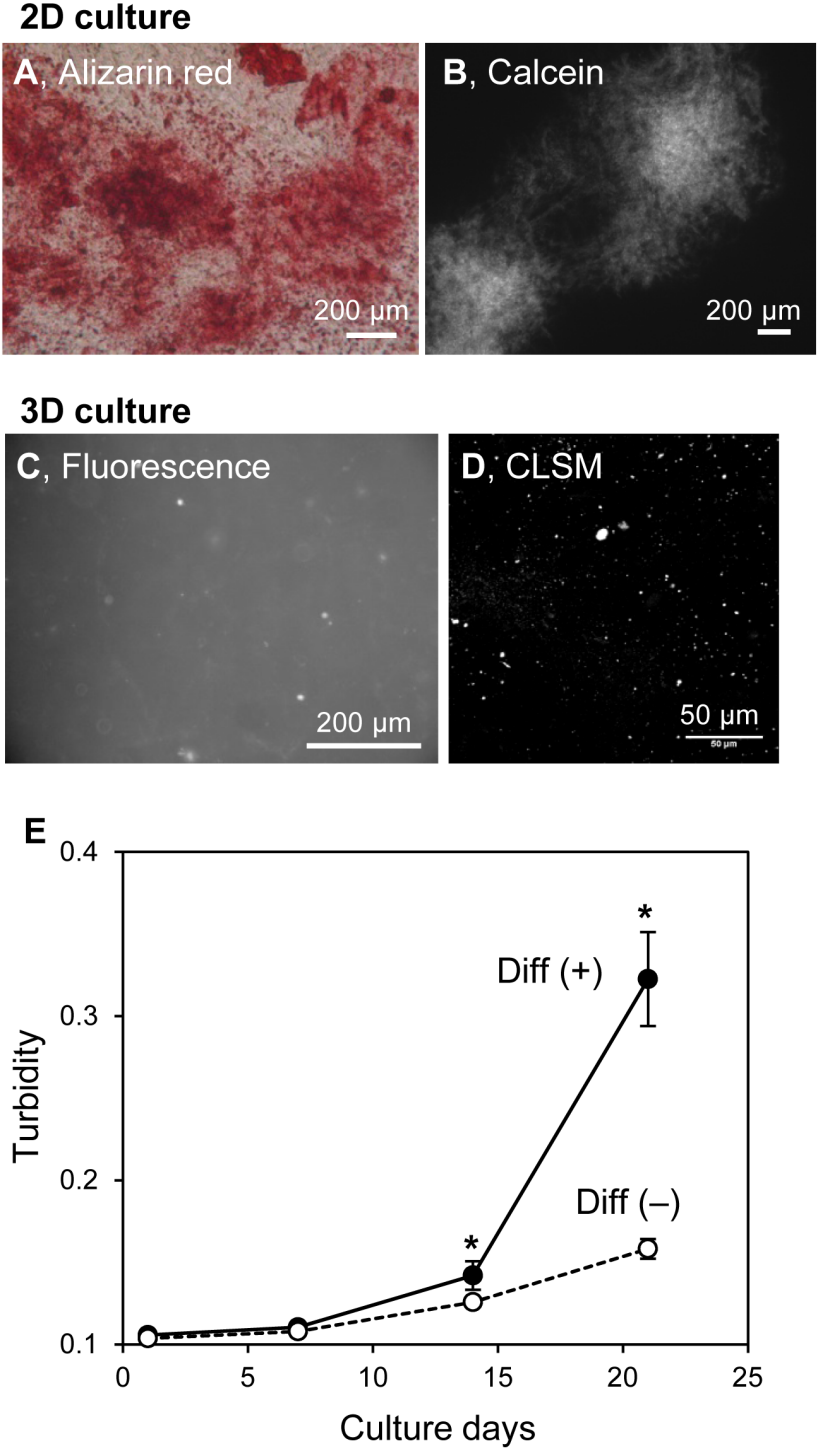
Mineralization of MC3T3-E1 cells in a 3D collagen gel. (A, B) Mineralized matrices of MC3T3-E1 cells on 2D culture plates. MC3T3-E1 cells were cultured under osteogenic differentiation conditions with calcein for 3 weeks. The calcein-labeled mineralized matrices formed were stained with Alizarin red S (A) and observed by fluorescence microscopy (B). Bar: 200 µm. (C, D) Fluorescence images of mineralized MC3T3-E1 cells in the collagen gel. MC3T3-E1 cells were cultured under osteogenic differentiation conditions with calcein for 3 weeks. The calcein-labeled mineralized matrices in the collagen gel were observed by conventional fluorescence microscopy (C) or CLSM with stereoscopy (D). The serial CLSM images obtained for the *Z*-axis (total 56 µm thickness) were superimposed. Bar: 200 µm (fluorescence) or 50 µm (CLSM). (E) Transition of turbidity of the collagen gel embedded with MC3T3-E1 cells. MC3T3-E1 cells were cultured with or without an osteogenic differentiation inducer in a 3D collagen gel. The gel volume was 500 µL per well of a 24-well culture plate. Transition of turbidity of the gel was measured using a plate reader. *p < 0.05 vs. turbidity of gel without osteogenic differentiation conditions (*N* = 4).

The structures of in vitro mineralized matrices were found to vary according to cell type (Kihara, 2015). The mineralization mechanism of HOS cells in 3D collagen gel and on 2D culture plates were similar. Thus, the formation of mineralized particles in hydrogels may be an HOS cell-specific phenomenon. MC3T3-E1 cells, known as osteogenic model cells, form large mineralized matrices under osteogenic differentiation conditions, including media containing ascorbic acid, β-GP, and dexamethasone (Kihara, 2015). Then, we cultured MC3T3-E1 cells on 2D culture plates and in 3D collagen gel under osteogenic conditions (Fig. 7). MC3T3-E1 cells formed large and hill-like mineralized matrices on 2D culture plates after 3 weeks of culture (Figs. 7A and 7B) and amorphous-shaped mineralized particles in 3D collagen gels, similar to those of HOS cells (Figs. 7C and 7D). The turbidity of the collagen gel, containing MC3T3-E1 cells with ascorbic acid, β-GP, and dexamethasone, increased rapidly after 14 days of culture; conversely, the turbidity of the gel containing MC3T3-E1 cells without these differentiation inducers, in which conditions the cells could not form mineralized matrices on the 2D culture plate, only increased a little (Fig. 7E). The gel turbidities, with and without differentiation inducers, after 3 weeks of culture were 0.32 and 0.16, respectively (Fig. 7E). Thus, the formation of mineralized particles in collagen gel is likely to be a common mineralization phenomenon of osteogenic cells.

## Discussion

In this study, we found that HOS and MC3T3-E1 cells formed amorphous mineralized particles in 3D hydrogel under mineralized matrix-forming conditions. These matrices were obviously different in shape from those formed by these osteogenic cells on 2D tissue culture plates. The shapes of mineralized matrices under 2D culture conditions varied with the type of osteogenic cell; HOS cells formed spherical mineralized matrices, ∼12 µm in diameter, and MC3T3-E1 cells form flat and large mineralized matrices, >200 µm in size, on 2D culture plates (Figs. 2 and 7). On the other hand, these cells, when cultured in 3D type I collagen gels, both formed mineralized particles (Figs. 1, 2 and 7), only under mineralization conditions, suggesting that the formation of mineralized particles in 3D collagen gels is, possibly, a common characteristic of osteogenic cells.

What exactly are the mineralized particles in collagen gel? The mineralized particles were not only formed in type I collagen gels but were also formed in the poly-ion-complexed peptide-based hydrogel, Panacea Gel (Fig. 5). The reconstituted type I collagen fibers have a D-periodic banded pattern which is formed by arranging the collagen molecules (Kadler et al., 1996), and osteogenic calcium is deposited in the space between the arranged collagen molecules (Hodge, 1989; White et al., 1977). On the other hand, Panacea Gel fiber does not show clear patterns and their based peptide is cationic, meaning that the peptide and the hydrogel do not have affinity for calcium ion (Nagai et al., 2012). We found that the mineralized particles were not simply calcium phosphate precipitates on hydrogel fibers. Furthermore, the formation of the mineralized particles was completely diminished by treatment with Foscarnet, an inhibitor of the sodium-phosphate cotransporter Pit-1 (Fig. 6) (Yoshiko et al., 2007). Thus, the formation of mineralized particles requires the biological process in cell membrane. The average size of the mineralized amorphous-shape particles was ∼1.7 µm and they were observed near the collagen gel-embedded cells (Fig. 2). The particles were too small to be considered calcified dead cells. Preferably it is considered that the mineralized particles are derived from vesicles secreted by osteogenic cells, which are anchored to hydrogel fibers. Osteogenic cells secrete matrix vesicles, which are nano-sized secretory vesicles from osteogenic cells and play a role in nuclear calcification and primary mineralization (Hasegawa et al., 2017). The primary mineralization crystal grows in and protrudes from the mineralized matrix and becomes calcospherulites (also referred to as calcified nodule and calcospherite) (Boyde & Sela, 1978; Hoshi, Ejiri & Ozawa , 2001; Midura et al., 2007). The average size of the calcospherulites mainly seen at the bone mineralization front is ∼0.5 µm (Hoshi, Ejiri & Ozawa , 2001; Midura et al., 2007). The calcospherulites gradually expand in the extracellular space (Hoshi, Ejiri & Ozawa , 2001). Furthermore, on initial mineralization in the matrix vesicle, inorganic phosphate is transported into the vesicle through Pit-1 (Hasegawa et al., 2017). Then, we assume that the mineralized particles formed by HOS and MC3T3-E1 cells in 3D hydrogels in our study are calcospherulites. Thus osteogenic cells can commonly form mineralized particles in 3D hydrogel, regardless of the type of hydrogel, under mineralization conditions.

Why were mineralized particles only observed in 3D hydrogel culture? If the mineralized particles are calcospherulites as mentioned above, mineralized particles should have also been observed in the 2D cell cultures, but these cells formed completely different mineralized matrices in 2D culture plates; HOS cells formed spherical and MC3T3-E1 cells formed large hill-like mineralized matrices (Figs. 2 and 7). However, the formation of mineralized matrices of HOS cells in 3D collagen gel and on 2D culture plates were both completely diminished by the inhibition of membrane transport of inorganic phosphate (Fig. 6). Thus, the mineralization processes and mechanisms of HOS cells in 2D and 3D culture conditions are considered the same. We previously showed that rat mesenchymal stem cells formed large and nodal mineralized matrices in 2D osteogenic differentiation culture conditions (Kihara et al., 2006; Kihara et al., 2004). Using FITC labelled exogenous type I collagen and fast Fourier transform (FFT) image processing, we found that they formed non-collagenous mineralization on the bottom of the culture dish and small calcium particles around the deposited type I collagen in the large and nodal mineralization matrices, but these different mineralization matrices could not be determined by the usual method of observation (Kihara et al., 2006). Thus, HOS and MC3T3-E1 cells also probably formed undetectable mineralized particles under 2D culture conditions. The mineralized particles formed under 3D culture conditions were a few µm-sized, amorphous-shaped and dispersed in 3D space (Fig. 2). Thus, if these cells formed mineralized particles under 2D culture conditions, it would be difficult to observe because the mineralized matrices formed by these cells under 2D culture conditions are larger and more condensed than mineralized particles (Figs. 1, 2 and 7). In fact, under 2D culture conditions, the peak size of small mineralized matrices formed by HOS cells was observed to be ∼1.9 µm (Fig. 2). Small-sized mineralized particles were also deposited under 2D culture conditions, but it was a little difficult to recognize these among the larger and more condensed mineralized matrices (Fig. 2).

What is the effect of 3D hydrogel culture for osteogenic cells? In 3D hydrogels, the cell environment is occupied by the hydrogel fibers, and then the cells interact with the fibers during the culture period. Type I collagen and Panacea Gel have a potential to increase and accelerate osteogenic cell activities (Ando et al., 2018; Kihara et al., 2006; Mizuno & Kuboki, 2001; Takeuchi et al., 1997; Xiao et al., 1998). Thus, osteogenic cells would form mineralized matrices earlier under 3D hydrogel conditions than 2D conditions as a result of interaction with the hydrogel fibers (Figs. 1 and 7). Furthermore, the cell densities in 3D and 2D culture conditions are very different; the cell density under 2D conditions is apparently higher than that under 3D conditions. These different cell densities may be related to the shape of the mineralized matrices. In high-density cell layers in 2D cultures, the secreted and mineralized matrix vesicles may easily develop into larger mineralized matrices, while, in 3D hydrogels, the secreted matrix vesicles are probably anchored on the cell surrounded hydrogel fibers. The anchored matrix vesicles are mineralized but will not develop into larger mineralized matrices as in 2D culture conditions. These hypotheses are for further study.

Various methods have been developed to evaluate the mineralization of osteogenic cells in a time-dependent manner. For example, using fluorescent dyes that bind calcium ions and a fluorescence scanner, the mineralization processes of HOS and mesenchymal stem cells can be monitored (Hirose et al., 2003; Uchimura et al., 2003). The intensities of the precipitated fluorescent dye almost correlate with the precipitated calcium contents (Maeda et al., 2007; Uchimura et al., 2003). In this study, we indicated that the turbidity of the cell-embedded collagen gel was roughly correlated with the precipitated calcium contents inside the gel and increased along with the formation of mineralized matrices (Figs. 3 and 4). Thus, we can estimate the progress of formation of the mineralized particles of osteogenic cells using hydrogel turbidity. Furthermore, mineralization was observed earlier in the 3D collagen gel than in the commonly used 2D culture. HOS cells, in particular, begin forming mineralized particles in 3D collagen gel within 3 days of culture. Thus, the HOS cell 3D collagen gel culture method under mineralization conditions is useful for in vitro mineralization research.

## Conclusions

In this study we developed a new model for in vitro formation of mineralized matrices by osteogenic cells using a 3D collagen gel culture. HOS and MC3T3-E1 cells formed mineralized particles, around 1.7 µm in size, in the collagen gel under mineralization conditions. These mineralized particles were morphologically different from the mineralized matrices that were formed by these cells on 2D tissue culture dishes. The precipitation process of the mineralized particles in the gel could be estimated by measuring the gel turbidity. The mineralized particles were not only formed in type I collagen gel but also formed in poly-ion complex hydrogel. The formation of mineralized particles by HOS cells was completely diminished by inhibition of the sodium-phosphate cotransporter. Thus, we assume that the mineralized particles are calcospherulites derived from calcified matrix vesicles, which are secreted by osteogenic cells. Hence, this 3D gel culture system of osteogenic cells is useful as a model for the formation of the mineralized particles, calcospherulites.

##  Supplemental Information

10.7717/peerj.7889/supp-1 Supplemental Information 1 Raw data of Figures Figs. 2B, 3, 4, 5A, 6B, and 7C

## Additional Information and Declarations

### Data Availability

The following information was supplied regarding data availability: The raw data is available as a Supplemental File.

## References

[ref-1] Al-Jallad HF, Nakano Y, Chen JL, McMillan E, Lefebvre C, Kaartinen MT (2006). Transglutaminase activity regulates osteoblast differentiation and matrix mineralization in MC3T3-E1 osteoblast cultures. Matrix Biology.

[ref-2] Ando K, Imagama S, Kobayashi K, Ito K, Tsushima M, Morozumi M, Tanaka S, Machino M, Ota K, Nishida K, Nishida Y, Ishiguro N (2018). Effects of a self-assembling peptide as a scaffold on bone formation in a defect. PLOS ONE.

[ref-3] Bell E, Ivarsson B, Merrill C (1979). Production of a tissue-like structure by contraction of collagen lattices by human fibroblasts of different proliferative potential in vitro. Proceedings of the National Academy of Sciences of the United States of America.

[ref-4] Boyde A, Sela J (1978). Scanning electron microscope study of separated calcospherites from the matrices of different mineralizing systems. Calcified Tissue Research.

[ref-5] Czekanska EM, Stoddart MJ, Richards RG, Hayes JS (2012). In search of an osteoblast cell model for in vitro research. European Cells and Materials.

[ref-6] Declercq H, Van den Vreken N, De Maeyer E, Verbeeck R, Schacht E, De Ridder L, Cornelissen M (2004). Isolation, proliferation and differentiation of osteoblastic cells to study cell/biomaterial interactions: comparison of different isolation techniques and source. Biomaterials.

[ref-7] Glimcher MJ (1976). Composition, structure and organizatin of bone and other mineralized tissues and the mechanism of calcification.

[ref-8] Hasegawa T, Yamamoto T, Tsuchiya E, Hongo H, Tsuboi K, Kudo A, Abe M, Yoshida T, Nagai T, Khadiza N, Yokoyama A, Oda K, Ozawa H, De Freitas PHL, Li M, Amizuka N (2017). Ultrastructural and biochemical aspects of matrix vesicle-mediated mineralization. Japanese Dental Science Review.

[ref-9] Hayashi T (1978). Time-dependent increase in the stability of collagen fibrils formed in vitro. I. Effects of pH and salt concentration on the dissolution of the fibrils. Journal of Biochemistry.

[ref-10] Hirose M, Kotobuki N, Machida H, Uchimura E, Ohgushi H (2003). Quantitative monitoring of in vitro mineralization process using fluorescent dyes. Key Engineering Materials.

[ref-11] Hodge AJ (1989). Molecular models illustrating the possible distributions of ’holes’ in simple systematically staggered arrays of type I collagen molecules in native-type fibrils. Connective Tissue Research.

[ref-12] Hoshi K, Ejiri S, Ozawa H (2001). Ultrastructural analysis of bone calcification by using energy-filtering transmission electron microscopy. Italian Journal of Anatomy and Embryology.

[ref-13] Kadler KE, Holmes DF, Trotter JA, Chapman JA (1996). Collagen fibril formation. Biochemical Journal.

[ref-14] Kale S, Biermann S, Edwards C, Tarnowski C, Morris M, Long MW (2000). Three-dimensional cellular development is essential for ex vivo formation of human bone. Nature Biotechnology.

[ref-15] Kartsogiannis V, Ng KW (2004). Cell lines and primary cell cultures in the study of bone cell biology. Molecular and Cellular Endocrinology.

[ref-16] Kihara T, Arai T, Arai F, Yamato M (2015). Three-dimensional mineralized tissue formation of cultured bone marrow stromal cells. Hyper bio assembler for 3D cellular systems.

[ref-17] Kihara T, Hirose M, Oshima A, Ohgushi H (2006). Exogenous type I collagen facilitates osteogenic differentiation and acts as a substrate for mineralization of rat marrow mesenchymal stem cells in vitro. Biochemical and Biophysical Research Communications.

[ref-18] Kihara T, Oshima A, Hirose M, Ohgushi H (2004). Three-dimensional visualization analysis of in vitro cultured bone fabricated by rat marrow mesenchymal stem cells. Biochemical and Biophysical Research Communications.

[ref-19] Kotobuki N, Matsushima A, Kato Y, Kubo Y, Hirose M, Ohgushi H (2008). Small interfering RNA of alkaline phosphatase inhibits matrix mineralization. Cell and Tissue Research.

[ref-20] Lian JB, Stein GS (1992). Concepts of osteoblast growth and differentiation: basis for modulation of bone cell development and tissue formation. Critical Reviews in Oral Biology and Medicine.

[ref-21] Lund AW, Stegemann JP, Plopper GE (2009). Inhibition of ERK promotes collagen gel compaction and fibrillogenesis to amplify the osteogenesis of human mesenchymal stem cells in three-dimensional collagen I culture. Stem Cells and Development.

[ref-22] Maeda M, Hirose M, Ohgushi H, Kirita T (2007). In vitro mineralization by mesenchymal stem cells cultured on titanium scaffolds. Journal of Biochemistry.

[ref-23] Maniatopoulos C, Sodek J, Melcher AH (1988). Bone formation in vitro by stromal cells obtained from bone marrow of young adult rats. Cell and Tissue Research.

[ref-24] Midura RJ, Vasanji A, Su X, Wang A, Midura SB, Gorski JP (2007). Calcospherulites isolated from the mineralization front of bone induce the mineralization of type I collagen. Bone.

[ref-25] Mizuno M, Kuboki Y (2001). Osteoblast-related gene expression of bone marrow cells during the osteoblastic differentiation induced by type I collagen. Journal of Biochemistry.

[ref-26] Murshid SA, Kamioka H, Ishihara Y, Ando R, Sugawara Y, Takano-Yamamoto T (2007). Actin and microtubule cytoskeletons of the processes of 3D-cultured MC3T3-E1 cells and osteocytes. Journal of Bone and Mineral Metabolism.

[ref-27] Nagai Y, Yokoi H, Kaihara K, Naruse K (2012). The mechanical stimulation of cells in 3D culture within a self-assembling peptide hydrogel. Biomaterials.

[ref-28] Ochi M, Uchio Y, Kawasaki K, Wakitani S, Iwasa J (2002). Transplantation of cartilage-like tissue made by tissue engineering in the treatment of cartilage defects of the knee. Journal of Bone and Joint Surgery. British Volume.

[ref-29] Ohgushi H, Dohi Y, Katuda T, Tamai S, Tabata S, Suwa Y (1996). In vitro bone formation by rat marrow cell culture. Journal of Biomedical Materials Research.

[ref-30] Parreno J, Buckley-Herd G, De-Hemptinne I, Hart DA (2008). Osteoblastic MG-63 cell differentiation, contraction, and mRNA expression in stress-relaxed 3D collagen I gels. Hart DA Mol Cell Biochem.

[ref-31] Rodan SB, Imai Y, Thiede MA, Wesolowski G, Thompson D, Bar-Shavit Z, Shull S, Mann K, Rodan GA (1987). Characterization of a human osteosarcoma cell line (Saos-2) with osteoblastic properties. Cancer Research.

[ref-32] Sonobe H, Mizobuchi H, Manabe Y, Furihata M, Iwata J, Hikita T, Oka T, Ohtsuki Y, Goto T (1991). Morphological characterization of a newly established human osteosarcoma cell line, HS-Os-1, revealing its distinct osteoblastic nature. Virchows Archive B: Cell Pathology Including Molecular Pathology.

[ref-33] Sudo H, Kodama HA, Amagai Y, Yamamoto S, Kasai S (1983). In vitro differentiation and calcification in a new clonal osteogenic cell line derived from newborn mouse calvaria. Journal of Cell Biology.

[ref-34] Takeuchi Y, Suzawa M, Kikuchi T, Nishida E, Fujita T, Matsumoto T (1997). Differentiation and transforming growth factor-beta receptor down-regulation by collagen-alpha2beta1 integrin interaction is mediated by focal adhesion kinase and its downstream signals in murine osteoblastic cells. Journal of Biological Chemistry.

[ref-35] Uchimura E, Machida H, Kotobuki N, Kihara T, Kitamura S, Ikeuchi M, Hirose M, Miyake J, Ohgushi H (2003). In-situ visualization and quantification of mineralization of cultured osteogenetic cells. Calcified Tissue International.

[ref-36] Veis A (2003). Mineralization in organic matrix frameworks. Reviews in Mineralogy and Geochemistry.

[ref-37] White SW, Hulmes DJ, Miller A, Timmins PA (1977). Collagen-mineral axial relationship in calcified turkey leg tendon by X-ray and neutron diffraction. Nature.

[ref-38] Xiao G, Wang D, Benson MD, Karsenty G, Franceschi RT (1998). Role of the alpha2-integrin in osteoblast-specific gene expression and activation of the Osf2 transcription factor. Journal of Biological Chemistry.

[ref-39] Yoneno K, Ohno S, Tanimoto K, Honda K, Tanaka N, Doi T, Kawata T, Tanaka E, Kapila S, Tanne K (2005). Multidifferentiation potential of mesenchymal stem cells in three-dimensional collagen gel cultures. Journal of Biomedical Materials Research Part A.

[ref-40] Yoshiko Y, Candeliere GA, Maeda N, Aubin JE (2007). Osteoblast autonomous Pi regulation via Pit1 plays a role in bone mineralization. Molecular and Cellular Biology.

